# Integrative Analysis of Energy Partition Patterns and Plasma Metabolomics Profiles of Modern Growing Pigs Raised at Different Ambient Temperatures

**DOI:** 10.3390/ani10111953

**Published:** 2020-10-23

**Authors:** Shuai Zhang, Hang Gao, Xiongkun Yuan, Junjun Wang, Jianjun Zang

**Affiliations:** State Key Laboratory of Animal Nutrition, College of Animal Science and Technology, China Agricultural University, Beijing 100193, China; zhangshuai16@cau.edu.cn (S.Z.); gaohang@cau.edu.cn (H.G.); 17812080828@163.com (X.Y.); wangjj@cau.edu.cn (J.W.)

**Keywords:** ambient temperature, energy partition pattern, modern growing pigs, plasma metabolomics profile

## Abstract

**Simple Summary:**

Most of the studies focusing on energy partition patterns of growing pigs and the related mechanisms raised at different ambient temperatures were carried out during the 1970s to the early 2000s. With the rapid developments in pig breeding, research updates on such topics concerning modern growing pigs have been absent in the last decade. Therefore, this study focused on the energy partition patterns of modern growing pigs with different bodyweights at gradient-ambient temperatures and investigated the underlying changes in plasma metabolites under such conditions. Modern growing pigs at heavier bodyweight were more sensitive to high temperatures on energy intake and partition. At high ambient temperatures, most of the identified metabolites altered are associated with decreased fatty acid oxidation, increased lipid formation, and increased protein degradation. The findings of this study will provide possible solutions to precisely formulate diets for modern growing pigs raised at different ambient temperatures, and can help to improve our knowledge on potential mechanisms of thermoregulation in modern pig breeds.

**Abstract:**

This study explores the energy partition patterns of modern growing pigs at 25 kg and 65 kg raised at gradient-ambient temperatures. It also investigates the underlying changes in plasma under such conditions, based on the integrative analysis of indirect calorimetry and non-target metabolomics profiling. Thirty-six barrows with initial BW of 26.4 ± 1.9 kg and 24 barrows with initial BW of 64.2 ± 3.1 kg were successively allotted to six respiration chambers with ambient temperatures set as 18 °C, 21 °C, 23 °C, 27 °C, 30 °C, and 32 °C, and four respiration chambers with ambient temperatures set as 18 °C, 23 °C, 27 °C, and 32 °C, respectively. Each pig was kept in an individual metabolic crate and consumed feed ad libitum, then transferred into the respiration chamber after a 7-day adaptation period for 5-day indirect calorimetry assay and 1-day fasting. As the ambient temperature increased from 18 °C to 32 °C, the voluntary feed intake, metabolizable energy intake, nitrogen intake, and retention, total heat production, and energy retention as a protein of growing pigs at 25 kg and 65 kg all linearly decreased (*p* < 0.05), with greater coefficients of variation for pigs at 65 kg when temperatures changed from 18 °C to 32 °C. The cortisol and thyroid hormone levels in the plasma of 25 kg pigs linearly decreased as the ambient temperature increased from 18 °C to 32 °C (*p* < 0.05), and 13 compounds were identified through metabolomics analysis, including up-regulated metabolites involved in fatty acid metabolism, such as adrenic acid and down-regulated metabolites involved in amino acid metabolism, such as spermidine at 32 °C. These results suggested that modern growing pigs at heavier bodyweight were more sensitive to high temperatures on energy intake and partition. Most of the identified metabolites altered at high ambient temperatures are associated with suppressed fatty acid oxidation and elevated lipogenesis and protein degradation.

## 1. Introduction

Moderate ambient temperature in the house is the prerequisite for animals to maintain health and performance. In the modern large-scaled and high-efficient livestock production system, high or low temperature is one of the major stresses that animals face. Especially in tropical countries where the average ambient temperature frequently exceeds 25 °C and temperate countries exposed to summer heatwaves, heat stress can greatly impair pigs’ performance and welfare, leading to large economic loss for the pig industry [1]. Therefore, it is vital to keep the ambient temperature stable and appropriate during the life cycle of pigs.

When the effective ambient temperature is below the lower critical temperature (LCT) of the thermoneutral zone, heat production (HP) increases in warm-blooded animals, such as pigs, to meet the additional energy requirement of thermoregulation, with more feed are consumed [2]. On the other hand, the feed intake, HP, and energy retention (RE) of pigs will decrease when the ambient temperature is above the upper critical temperature (UCT) of the thermoneutral zone, along with the reduced locomotion [3]. Those threshold temperatures vary depending on animal-related factors, such as genotype, bodyweight (BW), and physiological stage [1].

The research on the effects of ambient temperature on patterns of physiological changes in livestock started in the 1940s, applying an artificial climate warehouse to mimic the environment in animal houses. In pigs, many studies were conducted from the 1970s to the early 2000s focusing on energy partition patterns at different temperatures using the climate-controlled open-circuit respiratory chamber [4,5,6,7,8,9]. With the development of animal breeding, modern pigs have greatly improved productivity (e.g., highly daily gain and higher reproductivity), but their bodies become more sensitive to the adverse environment, such as changes in ambient temperatures [10]. Therefore, research was carried out in recent years concerning the alternations and alleviation strategies of modern pigs under heat or cold stress. However, there are few updates on patterns of energy balances in modern pigs at different ambient temperatures, especially in the last decade.

Indirect calorimetry is the traditional non-invasive approach to determine the HP of the subjects and has been widely used in livestock to monitor the energy partition patterns together with the analysis of feed intake and urine and fecal output [11]. The metabolic changes underlying the energy reactions have received more attention in recent years, which could directly reflect the regulation and adaptation of the animals to the surrounding conditions, and may provide novel diagnostic tools or biomarkers with high sensitivity towards the adverse effects [4]. Non-targeting metabolomics is an ideal approach to analyze the comprehensive changes in metabolites of the subjects, and has never been used to explore the relationships between metabolites alternation and the patterns of energy expenditure or substrate oxidation of growing pigs raised at various ambient temperatures.

Therefore, the objectives of this study were to determine the energy partition patterns of modern growing pigs with different BWs at gradient-ambient temperatures and to identify the underlying changes in energy metabolism under such conditions based on the integrative analysis of indirect calorimetry and non-target plasma metabolomics profiling.

## 2. Materials and Methods

The Institutional Animal Care and Use Committee of China Agricultural University (Beijing, China) approved all protocols (Approval Number: AW31080202-1) used in this experiment.

### 2.1. Equipment for Indirect Calorimetry

Six open-circuit respiration chambers (1.4 m × 2.7 m × 2.1 m) located in the FengNing Swine Research Unit of China Agricultural University (Academician Workstation in Chengdejiuyun Agricultural and Livestock Co., Ltd., Chengde, China) were used to conduct the indirect calorimetry, which was described in details in previous studies [12,13] except for temperature settings. The relative humidity was controlled at 70–75% (EE10-M1A6D1 Moisture/Temperature Measuring Transducer, E+E Elektronik, Austria), and the air velocity was controlled at 0.1 m/s(Gas Mass Flow Meters, Alicat Scientific Inc., Tucson, AZ, USA). Oxygen content was measured with a paramagnetic differential analyzer (Oxymat 6E, Siemens, Munich, Germany), whereas CO_2_ and CH_4_ contents were measured with infrared analyzers (Ultramat 6E, Siemens, Munich, Germany). The analyzers had a range of 19.5% to 21.0% for O_2_, 0% to 1% for CO_2_, and 0% to 0.1% for CH_4_, with a sensitivity of 1% of the measurement range. Gas concentrations in each chamber were measured at 5-min intervals. The ethanol combustion experiment was used to check the accuracy of the chamber in measuring gaseous exchange at the beginning of each trial.

### 2.2. Animals, Diets, and Experiment Design

Two animal trials were conducted in this study. In Trial 1, 36 healthy growing barrows (Duroc × Large White × Landrace) with initial BW of 26.4 ± 1.9 kg were successively allotted to six open-circuit respiration chambers with ambient temperatures setting as 18 °C, 21 °C, 23 °C, 27 °C, 30 °C, and 32 °C, respectively, according to a completely randomized design. In Trial 2, 24 healthy growing barrows (Duroc × Large White × Landrace) with initial BW of 64.2 ± 3.1 kg were successively allotted to four open-circuit respiration chambers (only four chambers were available during Trial 2) with ambient temperatures setting as 18 °C, 23 °C, 27 °C, and 32 °C, respectively, according to a completely randomized design. In both trials, six successive replicates were included in each ambient temperature treatment.

Two diets based on corn-soybean meal were formulated to meet or exceed the nutrient requirements for growing pigs at 25 kg and 65 kg according to the recommendations by NRC (2012) [14] and were applied in Trial 1 and 2, respectively. The analyzed chemical compositions of these two diets are shown in Table 1.

The specific experimental procedures were kept the same in both animal trials. In each period, pigs were kept individually in stainless-steel metabolism crates (1.2 m × 0.5 m × 0.6 m) equipped with feeders and low-pressure nipple drinkers and had a 7-d adaption period for crates and diets in the thermoneutral-controlled room (24 ± 1 °C). On day 8, pigs were weighed and moved into the chambers for five days to measure the gas exchanges and calculate the total heat production (THP). Based on the feed intake during adaption, the feed was supplied twice daily (08:30 and 15:30) ad libitum to pigs in chambers, and pigs had free access to water. For each pig, the total feces, urine output, and feed refusal were collected twice daily from 08:00 to 08:30, and from 15:00 to 15:30, when the production of CO_2_, CH_4_, and O_2_ consumption were expelled in the calculation of daily HP, to determine the energy intakes of each day. On day 13, pigs were weighed in the morning and deprived of feed and fasted for 24 h, and the total urine output was collected during the fasting period to determine the fasting heat production (FHP), as an estimation of the energy used for maintenance [12]. On the morning of day 14, pigs were moved out of the chambers and weighed again.

### 2.3. Sample Collection

Representative feed samples were obtained after manufacturing and stored at −20 °C until analysis. The daily urine output of each pig was collected into buckets with 50 mL 6 N HCl and sieved thereafter through multilayer gauze into plastic bottles and stored at −20 °C. The 5-day total urine collection and urine collection during the fasting period were quantified for each pig, respectively, and a subsample of 50 mL for each pig was saved from the thoroughly mixed 5-day total collection for further analysis. The daily feces output of each pig was collected into plastic bags and immediately stored at −20 °C, then the 5-day total feces collection was thawed, mixed, and weighed, and a subsample of 350 g for each pig was oven-dried for 72 h at 65 °C to calculate the moisture content. Feed samples and subsamples of dried feces were finely ground through a 1 mm sieve before chemical analysis.

On day 13 morning at 7:30 (16 h after the last meal), blood samples of pigs in Trial 1 were collected from vena cava into the heparinized tubes, and then were centrifuged (Biofuge22R; Heraeus, Hanau, Germany) at 3000× *g* for 10 min at 4 °C. The supernatants were transferred to storage tubes, frozen in liquid nitrogen, and stored at −80 °C for further assays.

### 2.4. Chemical Analysis and Calculation

All chemical analysis of samples was conducted in duplicates and repeated if the duplicates differed by more than 5%. The gross energy (GE) in feed, feces, and urine samples was determined using an isoperibol calorimeter (Parr 6400 Calorimeter, Moline, IL, USA) with a standard reference of benzoic acid. For feed samples, the contents of dry matter (DM), crude protein (CP), ether extract (EE), and ash were determined following the methods of AOAC (2007) [15]. For fecal samples, the contents of DM and CP were determined following the methods of AOAC (2007) [15]. For urine samples, the nitrogen (N) content was measured, according to Li et al. (2018) [13].

Average BW and average daily gain during the entire experiment were calculated for each pig in both trials. The dry matter intake (DMi) of each pig from day 8 to day 12 was calculated by the feed intake multiplied by the DM content of the diets. The GE intake was calculated as the product of actual DMi during the 5-day collection period and the GE values of the diets. The digestible energy (DE) intake and metabolizable energy intake (ME_i_) was calculated as the difference between GE intake and the energy loss in feces, and the difference between DE intake and the energy loss in urine and methane, respectively. Methane energy was calculated using the methane volume and a conversion factor of 39.4 kJ/L [16].

The average daily THP was calculated according to the following equation based on the gas exchanges (O_2_ consumption and productions of CO_2_ and CH_4_) during day 8 to day 12 recorded in 5 min intervals and then averaged and extrapolated to a 24 h period.

HP (kJ) = 16.18 × O_2_ (L) + 5.02 × CO_2_ (L) − 2.17 × CH_4_ (L) − 5.99 × urinary N (g) [16]. To exclude the effect of ME_i_, THP was also adjusted to the same ME_i_ level at 2.4 MJ ME/kg BW^0.6^/d. The FHP was calculated using the same equation, but the 24-h FHP was predicted from the 8 h HP after feed deprivation from 22:00 to 06:00 during the last day of each period, which was then extrapolated to the 24-h period [13]. The RE was calculated as the difference between ME_i_ and THP, while the RE as protein (RE_P_) was calculated as N retention (g/d) × 6.25 × 23.86 (kJ/g), and the RE as lipid (RE_L_) was calculated as the difference between RE and RE_P_ [13]. The net energy (NE) intake was calculated as the sum of RE and maintenance energy estimated by FHP [17].

The rates of DE: GE, ME: DE, and NE: ME were then calculated based on the calculations of GE, DE, ME, and NE intakes. The respiratory quotient (RQ) was calculated as the ratio between CO_2_ production and O_2_ consumption. All the energy balance indexes were present on the metabolic bodyweight (per kg BW^0.60^) basis.

### 2.5. Hormone and Biochemical Marker Assays in Serum

The concentrations of cortisol, insulin, triiodothyronine (T_3_), and thyroxine (T_4_) in the serum of pigs from Trial 1 were analyzed using the automatic radioimmunoassay counter (XH-6020, Xi’an Nuclear Instrumentation Factory, Xi’an, China) with corresponding commercial kits (Beijing Sino-UK Institute of Biological Technology, Beijing, China) following the manufacturer’s guides, while the activities of growth hormone (GH) and glucagon in the serum of pigs from Trial 1 were analyzed using commercial enzyme-linked immunosorbent assay (ELISA) kits (Beijing Sino-UK Institute of Biological Technology, Beijing, China) following the manufacturer’s guides. The levels of albumin, glutamic-pyruvic transaminase (ALT), glutamic oxalacetic transaminase (AST), globulin, high-density lipoprotein (HDL), low-density lipoprotein (LDL), total cholesterol (TC), triglyceride (TG), total protein, and urea in the serum of pigs from Trial 1 were determined using the automatic biochemical analyzer (7170, Hitachi Corp., Tokyo, Japan) with corresponding commercial kits (Nanjing Jiancheng Bioengineering Institute, Nanjing, China) following the manufacturer’s guides.

### 2.6. Non-Target Metabolomics Profiling in Plasma and Data Analysis 

Plasma samples from pigs kept at the ambient temperatures of 18 °C, 23 °C and 32 °C (defined as low, neutral, and high ambient temperature group, respectively) were used for non-target metabolomics profiling assay, followed the procedures described by Liu et al. (2018) with some modifications [18].

Briefly, 100 μL plasma samples was added into 400 μL ice-cold extraction mix (methanol: acetonitrile = 1:1, v:v). After vortexing for 10 s, the mixture was centrifuged (Eppendorf, Hamburg, Germany) at 15,000× *g* for 10 min at 4 °C to remove protein, and 400 μL supernatant was collected and evaporated to dryness using a vacuum concentrator (Concentrator PLUS, Eppendorf, Hamburg, Germany). The resulting dry residues were re-suspended in 200 μL recovery solutions (water: methanol = 4:1), vortexed, and centrifuged again at 15,000× *g* for 10 min at 4 °C. The supernatant was filtered through a 0.22 μm membrane and transferred to sampler vials to be analyzed on a UPLC-MS system (UPLC, ACQUITY UPLC H-Class PLUS BioSystem, Waters Corporation, Milford, MA, USA; MS, Q-Exactive, Thermo Fisher Scientific, Waltham, MA, USA) equipped with heated electrospray ionization (HESI) source. The UPLC separation was operated on a BEH C18 column (2.1 × 100 mm, 1.7 μm, Waters Corporation), with mobile phase comprised of 0.1% formic acid water solution (A) and 0.1% formic acid acetonitrile solution (B), flow rate set at 0.3 mL/min, column temperature set at 35 °C, and injection volume set as 5 μL. The MS analysis was performed in an electrospray ionization positive mode, and data were acquired with a full scan using a mass resolution of 70,000 and a scan range of 50 to 750 *m*/*z*. For MS/MS analysis, an isolation window of 2.0 *m*/*z* and a mass resolution of 17,500 were selected.

### 2.7. Statistical Analysis

For the metabolomics data, the software SIEVE 2.1 (Thermo Fisher Scientific, Waltham, MA, USA) and Progenesis QI (Nonlinear Dynamics, Waters Corporation, Milford, MA, USA) were used for raw data processing, including background subtraction, peak alignment, identification, and normalization with Pareto scaling to get the standardized relative abundance of the metabolites in different samples. Then principal components analysis (PCA) was conducted using SIMCA-P 13 software (Umetrics, Umea, Sweden), and one-way ANOVA was conducted using JMP Pro 14.3.0 (SAS Institute Inc., Carry, NC, USA) to compare the abundance of metabolites among different treatments. The metabolites with fold change >1.5, CV < 20%, and *p* < 0.05 were selected for further identification by comparison of the ion features in the experimental samples to the chemical standard entries in reference libraries (METLIN, HMDB, and KEGG) that included retention time, molecular weight (*m*/*z*), as well as their associated MS/MS spectra. Boxplot was plotted using the ggplot2 package in R software (http://cran.r-project.org/, version 4.0.2) to better illustrate the concentration changes of the compounds that identified with significant differences among the three treatments. These compounds were then imported into the module of pathway analysis in Metaboanalyst 3.0 (https://www.metaboanalyst.ca/MetaboAnalyst/upload/PathUploadView.xhtml) to generate the pathway topology analysis. The metabolic pathway with an impact value greater than 0.1 was characterized as the significantly relevant pathway [18].

For the other data, normality and homogeneity of variance were checked using the normal probability plot and residual plot from JMP Pro 14.3.0, and outliers were removed before further analyses. A statistical model, including ambient temperature as the only fixed effect and chamber as the random effect, was fitted using the Fit Model function in JMP Pro 14.3.0. Tukey’s HSD test was used to separate the least square means among treatments with significantly different effects. Linear and quadratic effects of increased ambient temperature on the responses were tested by polynomial contrast using the Fit Y by X function in JMP Pro 14.3.0.

Equations were developed to predict the voluntary feed intake (VFI, kg), ME_i_ (kJ/d), RE_P_ (kJ/d), and RE_L_ (kJ/d) using metabolic bodyweight (BW^0.6^, kg) and ambient temperature (°C) as predictors based on some modifications of the equations from Quiniou et al. (2000) [19]:

VFI or ME_i_ = a + b × BW^0.6^ + c × (BW^0.6^)^2^ + d × T + e × T^2^ + f × BW^0.6^ × T,

RE_P_ or RE_L_ = a + b × BW^0.6^ + c × (BW^0.6^)^2^ + d × T + e × T^2^ + f × BW^0.6^ × T + g × ME_i_,

where a, b, c, d, e, f, and g represent coefficients, and T represents ambient temperatures.

All the data obtained in Trial 1 and 2 were combined and used for model development. Covariance analysis was used to further confirm the quadratic effects of BW and T on response variables, and coefficients were estimated by Gauss-Newton analysis using the Nonlinear Modelling function in JMP Pro 14.3.0. For all the analyses, *p* < 0.05 were considered as significantly different.

## 3. Results

### 3.1. Energy Partition and N Balance in Pigs at 25 kg and 65 kg Raised at Different Ambient Temperatures

Energy partition patterns of modern growing pigs at 25 kg kept at 18 °C, 21 °C, 23 °C, 27 °C, 29 °C, and 32 °C and pigs at 65 kg kept at 18 °C, 23 °C, 27 °C, and 32 °C are presented in Table 2 and Table 3, respectively. Pigs stayed at 32 °C demonstrated lower average daily gain compared to those at other ambient temperatures at either bodyweight (*p* < 0.05). As the ambient temperature increased from 18 °C to 32 °C in both growing stages, the DMi, N intake, fecal N output, N retention, ME_i_, THP, and RE_P_ of growing pigs all showed linearly and quadratically decreased patterns (*p* < 0.05).

Specifically, for pigs at 25 kg, animals housed at 32 °C had lower DMi, N intake, N retention, ME_i_, THP, and RE_P_ compared with those raised at 18 °C, while pigs kept at 29 °C also had lower THP compared with those kept at 18 °C (*p* < 0.05). Besides, pigs raised at 18 °C and 21 °C exhibited greater fecal N output than those raised at 27 °C, and pigs housed at 23 °C exhibited greater RE_P_ than those housed at 32 °C (*p* < 0.05). As the ambient temperature increased from 18 °C to 32 °C, the RQ at fasting state linearly and quadratically increased, with pigs housed at 32 °C showing greater RQ during fasting state compared with those housed at 18 °C, 21 °C and 23 °C (*p* < 0.05).

On the other hand, for pigs at 65 kg, animals raised at 27 °C and 32 °C had lower DMi, N intake, N retention, ME_i_, THP, RE, and RE_L_ compared with those raised at 18 °C and 23 °C (*p* < 0.05). Moreover, pigs housed at 32 °C demonstrated lower fecal and urine N outputs and RE_P_ compared with those housed at 18 °C and 23 °C, lower RQ during fed state compared with those housed at 18 °C, 23 °C and 27 °C, lower NE/ME compared with those housed at 23 °C and 27 °C, but greater adjusted THP and methane energy/DE ratio compared to those housed at other ambient temperatures (*p* < 0.05). Otherwise, no significant changes were observed on other parameters related to energy balance and utilization at different ambient temperatures in both growth stages (*p* > 0.05).

The coefficients of variation when temperatures changed from 18 °C to 32 °C were used to compare the results between the two trials. For DMi, N retention, ME_i_, RE, RE_P_, and RE_L_, those coefficients of variation for pigs at 25 kg and 65 kg were 15.8 vs. 26.6, 18.2 vs. 20.4, 15.7 vs. 30.4, 26.2 vs. 46.1, 18.1 vs. 34.6 and 34.6 vs. 48.6, respectively, with pigs at 65 kg showing greater coefficients of variation for all these parameters.

Besides, models were developed in the current study to predict VFI, ME_i_, RE_P_, and RE_L_ using BW and ambient temperature as predictors, and are presented in Table 4.

### 3.2. Changes in Hormone and Biochemical Marker Levels in Serum of Pigs at 25 kg Raised at different Ambient Temperatures

The concentrations of hormone and biomedical markers in serum of modern growing pigs at 25 kg kept at 18 °C, 21 °C, 23 °C, 27 °C, 29 °C, and 32 °C are presented in Table 5. The levels of cortisol, T_3_, T_4_, HDL, and TC in pig serum showed patterns of linearly and quadratically decreased as the ambient temperature increased from 18 °C to 32 °C, with the T_4_ level at 18 °C greater than that at 27 °C, 29 °C and 32 °C, the HDL level at 18 °C greater than that at 27 °C and 29 °C, and the TC level at 18 °C, 21 °C and 23 °C greater than that at 27 °C (*p* < 0.05). The glucagon and AST levels in pig serum quadratically changed as the ambient temperature increased from 18 °C to 32 °C, with the AST level at 29 °C greater than that at 18 °C (*p* < 0.05), and the glucagon level at 27 °C tended to be the lowest among all the treatment groups (*p* = 0.054).

### 3.3. Metabolomics Profiles in Plasma of Pigs at 25 kg Raised at different Ambient Temperatures

**Figure 1:**
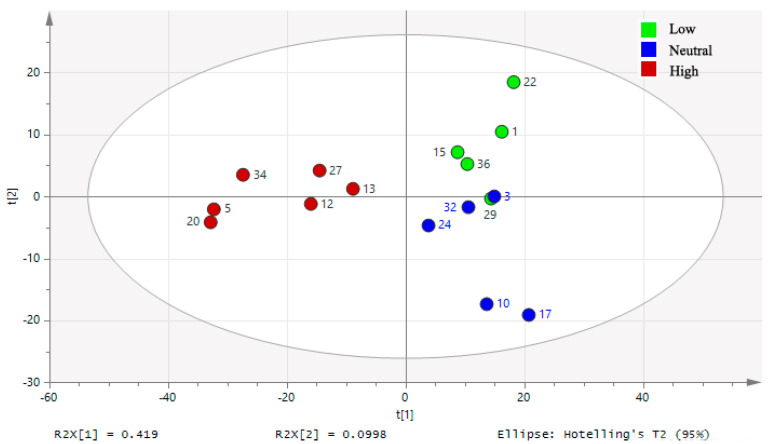
Principle component analysis (PCA) score plot demonstrating the separation of the plasma samples from pigs at 25 kg kept at different ambient temperatures. Low: 18 °C; Neutral: 23 °C; High: 32 °C. Each circle represents an individual plasma sample.

The PCA score plot showed that the plasma samples from pigs stayed at low (18 °C), neutral (23 °C), and high (32 °C) ambient temperatures clustered within the 95% confidence interval (Figure 1). The PC1 and PC2 accounted for 41.9% and 9.98% of the total variance of the data, respectively, indicating there was a major difference among the metabolomics profiles of the three treatment groups.

**Figure 2:**
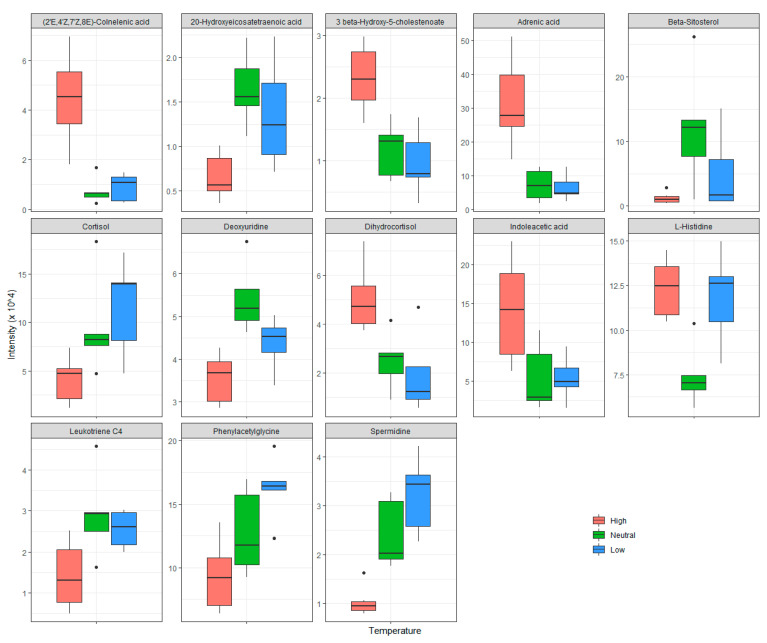
Identified compounds through metabolomics analysis with different intensities in plasma samples from pigs at 25 kg kept at different ambient temperatures. No statistical analysis was conducted in this figure. Low: 18 °C; Neutral: 23 °C; High: 32 °C.

A total of 13 compounds were identified with different intensities among the three treatment groups by comparing to the standard entries in reference libraries (Table 6), which include categories of amino acid, fatty acid, and steroid. To better illustrate the changes of those compounds in the treatment groups, data are visualized through boxplot in Figure 2. Compared to pigs stayed at the neutral temperature (23 °C) or low temperature (18 °C), the concentrations of (2’E, 4’Z, 7’Z, 8E)-colnelenic acid, 3-beta-hydroxy-5-cholestenoate, adrenic acid, dihydrocortisol, and indoleacetic acid all dramatically up-regulated, while the concentrations of 20-hydroxyeicosatetraenoic acid, beta-sitosterol, cortisol, deoxyuridine, leukotriene C4, phenylacetylglycine, and spermidine all greatly down-regulated in plasma of pigs stayed at the high temperature (32 °C). Besides, compared to pigs stayed at neutral temperature, the levels of beta-sitosterol markedly up-regulated, while the levels of (2’E, 4’Z, 7’Z, 8E)-colnelenic acid, cortisol, *L*-histidine, phenylacetylglycine, and spermidine slightly down-regulated in plasma of pigs stayed at the low temperature (18 °C).

According to the pathway analysis, the identified metabolites are mainly involved in pathways related to amino acid and lipid metabolism, including histidine metabolism, beta-alanine metabolism, tryptophan metabolism, phenylalanine metabolism, arachidonic acid metabolism, steroid hormone biosynthesis, alpha-linolenic acid metabolism, biosynthesis of unsaturated fatty acids, and steroid biosynthesis. Only the pathway of histidine metabolism had an impact value greater than 0.1 (impact value = 0.22), which is the cut-off value of relevance gained from the topology analysis.

## 4. Discussion

### 4.1. Effects of Ambient Temperature on Energy Partition and N Balance of Modern Growing Pigs

Pig are warm-blooded animals. At low ambient temperatures, increased feed intake and heat production can facilitate the maintenance of constant body temperature [20]. The criteria of “low” temperature are defined according to the thermoneutral zone and the LCT value, which was usually estimated by the following equation: LCT (°C) = 17.9 − 0.0375 × BW [14]. Based on the temperature settings in the current study, all pigs used in the animal trial were kept above the LCT. When the ambient temperature surpasses the UCT, the heat production of the pig body becomes a “burden”, and pigs can keep the body temperature fluctuate in a small range by increasing the evaporation heat loss and decreasing the heat production [20]. In the current study, we observed decreased Dmi and ME_i_ when the ambient temperature increased from 23 °C to 32 °C for pigs at both 25 kg and 65 kg, which were in accordance with the previous reports [1,2,3,4,5,6,7,8,9,19,20,21,22,23]. Due to the degradation of the sweat gland, the capacity of heat dissipation through the evaporation of pigs is limited [20]. Therefore, at high ambient temperatures, the feed intake of pigs will decrease dramatically to control their heat production. The impaired feed and energy intake may also attribute to the damaged gut morphology and function of pigs [24]. But increased THP adjusted into the same ME_i_ level was observed for pigs at 65 kg in the current study. Close and Mount (1971) also illustrated that the THP was the lowest during 20 °C to 25 °C, and gradually increased when the temperature reached 30 °C [21], which could be attributed to the extra energy expenditure used for the additional activity, such as more frequent breathing and heart rating to facilitate the heat dissipation [9]. The above conflict in heat production results may rely on the specific temperature range, BW, and feeding level of the pigs [22].

The environmental temperature could affect the partition of ME_i_, specifically, into maintenance energy, energy for thermoregulation, and energy for growth (the RE) in growing pigs. In the current study, no significant influence of ambient temperatures on maintenance energy (ME_m_, estimated by FHP) and the partial energy utilization efficiency of digestion (DE/GE) and metabolization (ME/DE) of pigs at 25 kg and 65 kg were observed. But decreased RQ during the fed state for pigs at 65 kg, and decreased RE_P_ for pigs at 25 kg and RE, RE_P_, and RE_L_ for pigs at 65 kg were observed when the environment temperature increased from 18 °C to 32 °C, reflecting reduced maintenance energy partition, and protein and lipid synthesis at high ambient temperature especially for pigs at heavier bodyweight. Close (1978) also demonstrated decreased ME_m_ from 723 to 469 kJ/kg BW^0.75^/d when the environmental temperature increased from 10 °C to 30 °C [23]. The declined partial energy efficiency estimated for protein deposition, and lipid deposition was also reported [23], which are similar to our observations, indicating that part of the energy was used to dissipate the heat produced during protein and lipid synthesis at high ambient temperature, leading to the decreased partial energy efficiency. This inference could also be supported by the decreased NE/ME ratio for pigs at 65 kg in the current study. Due to the absence of dietary energy gradient settings, the changes of partial efficiency for protein and lipid synthesis at different temperatures were not tested in the current study.

In the current study, we also focused on the effects of ambient temperatures on N balance. When the ambient temperature increased from 18 °C to 32 °C, the N intake, fecal N output, and N retention of pigs at both 25 kg and 65 kg all decreased, also reflecting the reduced protein synthesis at high temperatures. The environmental temperature had no influence on urine N output for pigs at 25 kg, but altered urine N output for pigs at 65 kg in our study, which was partially supported by Guo (2004) and Verstegen et al. (1973), who reported independence of urine N output with ambient temperatures [2,22]. Those discrepancies in results of urine N output could be mainly ascribed to the differences in the growing stages of pigs.

It has been widely proven that pigs at different growth stages have distinct patterns of energy partition as the ambient temperatures change, since the critical temperatures differ for pigs with different BW [14]. In the current study, we found the coefficients of variation for DMi, N retention, ME_i_, RE, RE_P_, and RE_L_ were greater for pigs at 65 kg than those at 25 kg, indicating that pigs at heavier bodyweight were more sensitive to high temperatures on energy intake and partition, which is in consistence with the conclusion from the meta-analysis conducted by Renaudeau et al. [25]. Therefore, the quadratic models, including the main effects of metabolic BW, ambient temperature, and their interaction effects, were used to fit the raw data in the current study and to predict the VFI, ME_i_, as well as RE_P_ and RE_L_.

Similar simulation equations have already been developed in some previous studies to model VFI and ME_i_ in pigs exposed to high ambient temperatures [8,19,22], which were published about 20 years ago and could be treated as reflections of old-genetic pigs raised at different ambient temperatures. Compared these curvilinear models with those developed in our study, it can be seen that pigs in the current study, as a reprehensive of the modern genotypes, were more sensitive to high temperatures on VFI and ME_i_ reflected by more rapidly-changing tangent slopes of the curvilinear, especially at heavier BW. This observation was also supported by Renaudeau et al. (2011), who analyzed the effects of high temperature on feed intake of pigs with different BW using meta-analysis based on 86 trials and 202 temperature treatments published from 1970 to 2009, and found that the effect of increased temperature was greater in more contemporary works [25]. This is reasonable because the genetic selection of modern pigs mainly aimed at improving the quality and efficiency of lean tissue growth, neglecting the capacity dealing with heat stress.

One flaw of the models developed in the current study is that the BW data collected for simulation only came from two growth stages, and the narrow range of BW may produce bias when predicting the responses of pigs with other BWs.

### 4.2. Effects of Ambient Temperature on Hormone and Biochemical Markers in Serum of Modern Growing Pigs

As the ambient temperature increased from 18 °C to 32 °C, there was linearly decreased cortisol, T_3_, T_4_, HDL, and TC levels, linearly increased AST level, and quadratically changed glucagon level in serum of 25 kg pigs in the current study. Similar changes in physiological and biomedical parameters in plasma of growing pigs under heat stress were also reported previously [26,27]. The decreased cortisol content in plasma may indicate improved protein degradation to provide amino acids for gluconeogenesis [27]. The decreased cortisol level and increased glucagon and AST levels in plasma may also act as indicators for the stress reaction of pigs under extreme environmental conditions [27]. In addition, the depressed thyroid hormone (T_3_ and T_4_) levels have been associated with lowered metabolic heat production in pigs kept at high environmental temperatures [27], which was also supported by the decreased THP in pigs when the ambient temperature increased from 18 °C to 32 °C in the current study. Meanwhile, a reduction in HDL and TC, indicating the decreased β-oxidation of fat for energy purpose and favored hepatic synthesis of triglycerides, could also support the depressed plasma thyroid hormone levels in pigs reared at high ambient temperature [26].

### 4.3. Effects of Ambient Temperature on Plasma Metabolomics Profiles of Modern Growing Pigs

To simplify the analysis, only plasma samples from 18 °C, 23 °C and 32 °C were used for metabolomics analysis in the current study. Among the 13 compounds identified, more compounds showed significant changes in pigs at high ambient temperature than those at relatively low ambient temperature, and most of the metabolites in plasma only exhibited slightly changes in pigs at 18 °C compared to that at 23 °C, indicating that modern growing pigs are more sensitive to heat stress reflected by the plasma metabolites. Another reason may be that the “low” temperature settings in the current study may not produce cold stress, since it is above the LCT defined by NRC (2012) [14].

At high ambient temperature, most of the metabolites with up-regulated expressions belong to fatty acids ((2’E, 4’Z, 7’Z, 8E)-colnelenic acid, 3-beta-hydroxy-5-cholestenoate, and adrenic acid), or involve in pathways related to lipid metabolism or fatty acid metabolism (dihydrocortisol), indicating elevated lipogenic pathways and suppressed fatty acid oxidation. In agreement with our findings, many previous studies also showed that heat stress could increase lipid retention by enhancing lipogenesis and inhibiting lipolysis in pigs [26,28,29]. Qu and Ajuwon (2018) also detected greater serum linoleic and total polyunsaturated fatty acids levels using metabolomics in pigs stayed at 35 °C [30], which agrees with the observations in our study. These changes in lipid and fatty acid metabolism are also reflected by the reduced HDL, TC, and thyroid hormone levels in serum in the current study. On the other hand, the metabolites with down-regulated expressions at 32 °C are involved in amino acid metabolism (phenylacetylglycine and spermidine), or lipid metabolism (20-hydroxyeicosatetraenoic acid, beta-sitosterol, and leukotriene C4), which may indicate the enhanced protein degradation and gluconeogenesis, as well as the depressed lipolysis, and was partially supported by some previous studies [31,32]. Interestingly, cortisol was also identified in the metabolomics analysis with a down-regulated level at high temperature, consistent with the results in the plasma hormone assay, and can be reflected by the decreased RE_P_ in the energy partition pattern and the decreased N retention pattern in the current study. Furthermore, the suppressed fatty acid oxidation and elevated lipogenesis and protein degradation at high ambient temperature could also be related to the impaired zootechnical performance, especially the decreased feed intake, which were also observed in pigs during fasting [18].

### 4.4. Limitations and Prospects of the Current Study

The originality of the current study was the simultaneous measurement of heat production and metabolic indicators in pigs raised at different ambient temperatures. Unfortunately, it has been done only in pigs at 25 kg, due to the budget limitation. According to our results, pigs at 65 kg were more sensitive to heat stress, thus it will be more pertinent to conduct the metabolomics analysis on heavier pigs under heat stress in the future. Moreover, some physiological indicators of heat stress, such as respiration rate, body temperature, and pig behaviors that could contribute to variations of heat production and energy partition, can be complemented in further study. When the results of the current study were applied to practical situations, the specificity of this study should be paid attention to. For instance, the current study demonstrated the short-term response of pigs to high ambient temperatures, considering the absence of adaptation to the temperature changes before the measurements. Unlike the variable environmental temperatures in practice, the temperatures in respiration chambers are constant over the day. In addition, the single-housing and the metabolic cage housing conditions were all different from settings in some previous studies reporting heat stress responses of pigs, and of course, the practical conditions.

## 5. Conclusions

The current study suggested modern growing pigs at heavier bodyweight were more sensitive to high temperatures on energy intake and partition. The integrative analysis of indirect calorimetry and metabolomics profiling revealed that the decreased energy intake and total heat production at 32 °C are associated with suppressed fatty acid oxidation and elevated lipogenesis and protein degradation, reflected by biomarkers, including up-regulated adrenic acid and down-regulated cortisol levels in plasma. Our findings provide possible solutions to precisely formulate diets for modern growing pigs raised at different ambient temperatures, and can help to improve our knowledge on potential mechanisms of thermoregulation in modern pig breeds.

## Figures and Tables

**Table 1:** Ingredient compositions and analyzed chemical components of the complete diets fed to barrows at different growth stages used in Trial 1 and Trial 2 (as-fed basis).

Items	Growth Stage	
	25 kg	65 kg
Ingredients, %		
Corn	76.93	80.19
Soybean meal	20.00	17.00
Dicalcium phosphate	0.90	0.80
Limestone	0.75	0.65
Sodium chloride	0.35	0.35
Premix ^1^	0.50	0.50
Lysine-HCl	0.41	0.37
DL-Methionine	0.11	0.09
L-Threonine	0.02	0.02
L-Tryptophan	0.03	0.03
Analyzed nutrient levels, %		
Dry matter	89.50	89.60
Gross energy, MJ/kg	16.38	16.67
Crude protein	17.78	16.20
Ether extract	2.89	2.57
Ash	4.72	3.34
Calculated nutrient levels, %		
Digestible energy, MJ/kg	14.26	14.27
Standardized ileal digestible lysine	0.99	0.89
Standardized ileal digestible methionine + cysteine	0.58	0.53

^1^ Premix supplied the following quantities per kilogram of complete diet for pigs at 25 kg: Vitamin A, 9000 IU; vitamin D_3_, 3000 IU; vitamin E, 60 IU; vitamin K_3_, 3 mg; vitamin B_12_, 30 μg; riboflavin, 5.5 mg; pantothenic acid, 15 mg; niacin, 40 mg; choline chloride, 550 mg; folacin, 0.8 mg; thiamine 1.5 mg; pyridoxine 3 mg; biotin, 100 μg; Mn, 40 mg (MnO); Fe, 100 mg (FeSO_4_·H_2_O); Zn, 100 mg (ZnO); Cu, 150 mg (CuSO_4_·5H_2_O); I, 0.3 mg (KI); Se, 0.3 mg (Na_2_SeO_3_), and supplied the following quantities per kilogram of complete diet for pigs at 65 kg: Vitamin A, 5500 IU; vitamin D_3_, 2200 IU; vitamin E, 30 IU; vitamin K_3_, 2.2 mg; vitamin B_12_, 28 μg; riboflavin, 4 mg; pantothenic acid, 14 mg; niacin, 30 mg; choline chloride, 400 mg; folacin, 0.7 mg; thiamine 1.5 mg; pyridoxine 3 mg; biotin, 60 μg; Mn, 40 mg (MnO); Fe, 75 mg (FeSO_4_·H_2_O); Zn, 75 mg (ZnO); Cu, 100 mg (CuSO_4_·5H_2_O); I, 0.3 mg (KI); Se, 0.3 mg (Na_2_SeO_3_).

**Table 2:** Nitrogen and energy balance of barrows at 25 kg kept at different ambient temperatures ^1^.

Items	Ambient Temperature (°C)						SEM	*p*-Value ^5^		
	18	21	23	27	29	32		ANOVA	Linear	Quadratic
Average bodyweight, kg	28.7	28.7	27.0	28.7	28.8	28.7	0.8	0.587	0.709	0.622
Average daily gain, g	913 ^a^	860 ^a,b^	867 ^a,b^	763 ^a,b^	927 ^a^	529 ^b^	79	0.030	0.024	0.030
Dry matter intake, kg/d	1.32 ^a^	1.24 ^a,b^	1.21 ^a,b^	1.05 ^a,b^	1.16 ^a,b^	0.99 ^b^	0.06	0.030	<0.001	0.002
Nitrogen balance, g/d										
Intake	41.9 ^a^	39.3 ^a,b^	38.4 ^a,b^	33.4 ^a,b^	36.8 ^a,b^	31.6 ^b^	2.1	0.030	<0.001	0.002
Fecal output	6.7 ^a^	6.6 ^a^	5.9 ^a,b^	4.3 ^b^	4.6 ^a,b^	4.8 ^a,b^	0.5	0.004	<0.001	<0.001
Urine output	5.4	4.6	3.2	4.9	6.0	3.8	0.9	0.327	0.867	0.926
Retention	29.8 ^a^	28.0 ^a,b^	29.2 ^a,b^	24.7 ^a,b^	26.2 ^a,b^	21.7 ^b^	1.7	0.040	<0.001	0.003
Energy balance, kJ/kg BW^0.6^/d										
ME intake	2699 ^a^	2510 ^a,b^	2555 ^a,b^	2197 ^a,b^	2426 ^a,b^	2035 ^b^	133	0.025	<0.001	0.004
Total heat production	1133 ^a^	1093 ^a,b^	1075 ^a,b^	1000 ^a,b^	947 ^b^	925 ^b^	55	0.004	0.002	0.011
Total heat production adjusted ^2^	1010	1063	1034	1123	954	1069	87	0.629	0.875	0.953
Fasting heat production (FHP)	716	713	628	713	666	698	49	0.809	0.758	0.748
Energy retention (RE)	1565	1417	1480	1202	1478	1145	144	0.193	0.059	0.172
RE_P_^3^	594 ^a^	558 ^a,b^	602 ^a^	491 ^a,b^	521 ^a,b^	431 ^b^	33	0.013	<0.001	0.001
RE_L_^4^	971	860	879	715	957	714	121	0.409	0.220	0.471
Respiratory quotient										
Fed state	1.07	1.09	1.09	1.08	1.05	1.12	0.03	0.576	0.520	0.635
Fast state	0.80 ^b^	0.80 ^b^	0.80 ^b^	0.83 ^a,b^	0.83 ^a,b^	0.87 ^a^	0.02	0.025	0.003	0.007
Energy utilization, %										
DE/GE	84.9	84.3	84.9	86.7	87.6	84.9	1.1	0.293	0.211	0.307
Urine energy/DE	1.0	1.1	1.1	1.2	1.4	1.3	0.2	0.709	0.114	0.283
Methane energy/DE	0.35	0.42	0.38	0.50	0.40	0.43	0.07	0.829	0.426	0.559
ME/DE	98.6	98.5	98.6	98.3	98.2	98.5	0.2	0.647	0.274	0.411
NE/ME	85.1	85.4	83.4	84.1	88.7	86.0	2.3	0.577	0.441	0.709

^1^ ANOVA, analysis of variance; DE, digestible energy; GE, gross energy; ME, metabolizable energy; NE, net energy. ^2^ Total heat production adjusted = Total heat production was adjusted to the same ME intake at 2.4 MJ ME/kg BW^0.6^/d. ^3^ RE_P_ = Energy retention as protein (kJ/kg BW^0.6^/d) = N retention (g) × 6.25 × 23.86 (kJ/g)/BW^0.6^. ^4^ RE_L_ = Energy retention as fat (kJ/kg BW^0.6^/d) = [RE (kJ) − energy retention as protein (kJ)]/BW^0.6^. ^5^ Linear and Quadratic represents the polynomial contrasts analysis on linear and quadratic effects of the ambient temperatures. ^a,b^ Within a row means with different superscript letters differ at *p* < 0.05.

**Table 3:** Nitrogen and energy balance of barrows at 65 kg kept at different ambient temperatures ^1^.

Items	Ambient Temperature (°C)				SEM	*p*-Value ^5^		
	18	23	27	32		ANOVA	Linear	Quadratic
Average bodyweight, kg	69.6	67.0	69.2	70.8	1.2	0.226	0.306	0.134
Average daily gain, g	1040 ^a,b^	1107 ^a^	1187 ^a^	698 ^b^	119	0.064	0.016	0.004
Dry matter intake, kg/d	2.29 ^a^	2.28 ^a^	1.71 ^b^	1.22 ^c^	0.09	<0.001	<0.001	<0.001
Nitrogen balance, g/d								
Intake	72.7 ^a^	72.5 ^a^	54.3 ^b^	38.8 ^c^	2.8	<0.001	<0.001	<0.001
Fecal output	11.2 ^a^	8.9 ^a,b^	6.9 ^b,c^	5.0 ^c^	0.7	<0.001	<0.001	<0.001
Urine output	21.1 ^a^	12.3 ^a,b^	12.2 ^a,b^	6.5 ^b^	2.2	0.003	<0.001	<0.001
Retention	39.5 ^a,b^	47.2 ^a^	35.2 ^b,c^	29.2 ^c^	2.2	<0.001	0.001	0.003
Energy balance, kJ/kg BW^0.6^/d								
ME intake	2774 ^b^	3292 ^a^	2177 ^c^	1466 ^d^	109	<0.001	<0.001	<0.001
Total heat production	1203 ^a^	1164 ^a^	1000 ^b^	898 ^b^	43	<0.001	<0.001	<0.001
Total heat production adjusted ^2^	1053 ^b^	848 ^c^	1107 ^b^	1423 ^a^	60	<0.001	0.011	<0.001
Fasting heat production (FHP)	734	731	658	727	51	0.638	0.699	0.703
Energy retention (RE)	1572 ^b^	2128 ^a^	1177 ^c^	568 ^d^	104	<0.001	<0.001	<0.001
RE_P_^3^	567 ^a,b^	725 ^a^	453 ^b,c^	296 ^c^	44	<0.001	0.004	<0.001
RE_L_^4^	1005 ^b^	1403 ^a^	724 ^c^	365 ^d^	75	<0.001	<0.001	<0.001
Respiratory quotient								
Fed state	1.09 ^a^	1.09 ^a^	1.06 ^a^	1.00 ^b^	0.01	<0.001	0.002	0.001
Fast state	0.80	0.81	0.81	0.83	0.01	0.224	0.122	0.266
Energy utilization, %								
DE/GE	86.2	89.3	88.8	86.8	0.84	0.099	0.999	0.030
Urine energy/DE	1.6	1.1	1.6	2.1	0.3	0.147	0.330	0.097
Methane energy/DE	0.46 ^b^	0.46 ^b^	0.75 ^a,b^	0.85 ^a^	0.08	0.006	0.001	0.004
ME/DE	97.9	98.5	97.7	97.3	0.3	0.079	0.130	0.069
NE/ME	72.0 ^b,c^	79.4 ^a^	72.7 ^b^	66.5 ^c^	2.1	<0.001	0.063	0.003

^1^ ANOVA, analysis of variance; DE, digestible energy; GE, gross energy; ME, metabolizable energy; NE, net energy. ^2^ Total heat production adjusted = Total heat production was adjusted to the same ME intake at 2.4 MJ ME/kg BW^0.6^/d. ^3^ RE_P_ = Energy retention as protein (kJ/kg BW^0.6^/d) = N retention (g) × 6.25 × 23.86 (kJ/g)/BW^0.6^. ^4^ RE_L_ = Energy retention as fat (kJ/kg BW^0.6^/d) = [RE (kJ) − energy retention as protein (kJ)]/BW^0.6^. ^5^ Linear and Quadratic represents the polynomial contrasts analysis on linear and quadratic effects of the ambient temperatures. ^a–d^ Within a row means with different superscript letters differ at *p* < 0.05.

**Table 4:** Prediction equations for voluntary feed intake, metabolizable energy intake, energy retention as protein, and energy retention as lipid using body weight and ambient temperature as predictors ^1^.

No.	Models	Coefficients Estimation ^2^							RMSE	R^2^	*p*-Value
		a	b	c	d	e	f	g			
1	VFI = a + b × BW^0.6^ + c × (BW^0.6^)^2^ + d × T + e × T^2^ + f × BW^0.6^ × T	−7.72	1.54	−0.059	0.15	−0.0022	−0.0088	/	0.18	0.86	<0.001
2	ME_i_ = a + b × BW^0.6^ + c × (BW^0.6^)^2^ + d × T + e × T^2^ + f × BW^0.6^ × T	−9242.45	2042.05	−91.95	242.32	−4.56	−8.33	/	316.30	0.67	<0.001
3	RE_P_ = a + b × BW^0.6^ + c × (BW^0.6^)^2^ + d × T + e × T^2^ + f × BW^0.6^ × T + g × ME_i_	−233.38	−67.28	3.16	39.58	−0.76	−0.26	0.26	61.66	0.86	<0.001
4	RE_L_ = a + b × BW^0.6^ + c × (BW^0.6^)^2^ + d × T + e × T^2^ + f × BW^0.6^ × T + g × ME_i_	−465.10	8.10	−1.03	−53.73	1.34	0.67	0.69	134.58	0.85	<0.001

^1^ BW, bodyweight (kg); ME_i_, metabolizable energy intake (kJ/kg BW^0.6^/d); RE_L_, energy retention as lipid (kJ/kg BW^0.6^/d); RE_P_, energy retention as protein (kJ/kg BW^0.6^/d); RMSE, root mean square error; T, ambient temperature (°C); VFI, voluntary feed intake (kg). ^2^ Coefficients are estimated using Gauss-Newton analysis.

**Table 5:** Hormone and biochemical markers levels in serum of barrows at 25 kg kept at different ambient temperature ^1^.

Items	Ambient Temperature (°C)						SEM	*p*-Value ^2^		
	18	21	23	27	29	32		ANOVA	Linear	Quadratic
Cortisol, ng/mL	61.8	67.3	61.6	59.2	47.3	36.9	7.9	0.058	0.006	0.010
Glucagon, pg/mL	89.6	83.9	76.6	63.6	83.6	98.1	6.7	0.054	0.673	0.012
Growth hormone, ng/mL	4.53	5.23	6.56	4.06	4.97	5.97	0.98	0.571	0.707	0.930
Insulin, µIU/mL	8.69	8.00	8.68	7.59	7.22	8.09	0.54	0.470	0.171	0.335
Thyroxine, ng/mL	58.6 ^a^	46.2 ^a,b^	45.7 ^a,b^	41.3 ^b,c^	33.6 ^b,c^	31.0 ^c^	3.7	<0.001	<0.001	<0.001
Triiodothyronine, ng/mL	0.63	0.62	0.59	0.55	0.59	0.52	0.03	0.061	0.010	0.039
Albumin, µmol/L	35.7	35.8	35.4	33.8	35.0	37.2	1.0	0.303	0.661	0.212
ALT, U/L	61.2	53.8	60.4	50.5	55.5	56.0	3.3	0.337	0.244	0.309
AST, U/L	35.2 ^b^	36.8 ^a,b^	48.5 ^a,b^	54.3 ^a,b^	55.5 ^a^	39.2 ^a,b^	4.6	0.013	0.082	0.005
Globulin, µmol/L	34.1	31.6	37.2	35.1	34.9	33.0	1.3	0.128	0.937	0.438
HDL, mmol/L	0.55 ^a^	0.49 ^a,b,c^	0.53 ^a,b^	0.40 ^c^	0.43 ^b,c^	0.46 ^a,b,c^	0.03	0.004	0.004	0.006
LDL, mmol/L	1.55	1.36	1.60	1.34	1.45	1.32	0.07	0.052	0.064	0.168
Total cholesterol,mmol/L	2.47 ^a^	2.30 ^a^	2.40 ^a^	2.05 ^b^	2.25 ^a,b^	2.26 ^a,b^	0.05	0.002	0.017	0.007
Triglyceride, mmol/L	0.54	0.44	0.48	0.54	0.49	0.40	0.04	0.134	0.198	0.351
Total protein, g/L	69.8	67.4	68.8	71.5	69.1	70.2	2.1	0.845	0.556	0.834
Urea, mmol/L	4.21	3.84	4.50	3.76	3.99	4.23	0.18	0.306	0.863	0.380

^1^ ALT, glutamic-pyruvic transaminase; AST, glutamic oxalacetic transaminase; ANOVA, analysis of variance; HDL, high-density lipoprotein; LDL, low-density lipoprotein. ^2^ Linear and Quadratic represents the polynomial contrasts analysis on linear and quadratic effects of the ambient temperatures. ^a–c^ Within a row means with different superscript letters differ at *p* < 0.05.

**Table 6:** Metabolites with significantly different concentrations in plasma of barrows at 25 kg kept at different ambient temperatures.

No.	Compounds	*m*/*z*	Formula	Fold Change ^1^			Pathway
				High/Neutral	Low/Neutral	High/Low	
1	(2’E,4’Z,7’Z,8E)-Colnelenic acid	275.1994	C_18_H_28_O_3_	4.97	0.84	5.91	alpha-Linolenic acid metabolism
2	20-Hydroxyeicosatetraenoic acid	303.2305	C_20_H_32_O_3_	0.48	1.21	0.40	Arachidonic acid metabolism
3	3-beta-Hydroxy-5-cholestenoate	399.3242	C_27_H_44_O_3_	2.39	1.22	1.96	Primary bile acid biosynthesis
4	Adrenic acid	355.2614	C_22_H_36_O_2_	4.83	1.11	4.35	Biosynthesis of unsaturated fatty acids
5	Beta-Sitosterol	397.3812	C_29_H_50_O	0.24	2.36	0.10	Steroid biosynthesis
6	Cortisol	363.2152	C_21_H_30_O_5_	0.35	0.82	0.43	Steroid hormone biosynthesis
7	Deoxyuridine	251.0629	C_9_H_12_N_2_O_5_	0.81	1.24	0.66	Pyrimidine metabolism
8	Dihydrocortisol	382.2571	C_21_H_32_O_5_	2.62	1.30	2.02	Steroid hormone biosynthesis
9	Indoleacetic acid	176.0700	C_10_H_9_NO_2_	2.61	1.01	2.59	Tryptophan metabolism
10	*L*-Histidine	156.0763	C_6_H_9_N_3_O_2_	1.04	0.63	1.66	Histidine metabolism /beta-Alanine metabolism/Aminoacyl-tRNA biosynthesis
11	Leukotriene C4	313.6596	C_30_H_47_N_3_O_9_S	0.56	1.14	0.49	Arachidonic acid metabolism
12	Phenylacetylglycine	211.1070	C_10_H_16_N_2_O_4_	0.57	0.79	0.73	Phenylalanine metabolism
13	Spermidine	146.1647	C_7_H_19_N_3_	0.32	0.75	0.43	beta-Alanine metabolism/Arginine and proline metabolism /Glutathione metabolism

^1^ Fold change represents ratios of the normalized intensity of the metabolites in plasma of growing pigs at two different ambient temperatures. The low, ambient temperature at 18 °C; Neutral, ambient temperature at 23 °C; High, ambient temperature at 32 °C. Fold change >1 represents the metabolite was up-regulated, and fold change <1 represents the metabolite was down-regulated.
